# Correction: Statistical segmentation model for accurate electrode positioning in Parkinson’s deep brain stimulation based on clinical low-resolution image data and electrophysiology

**DOI:** 10.1371/journal.pone.0302985

**Published:** 2024-04-29

**Authors:** Igor Varga, Eduard Bakstein, Greydon Gilmore, Jaromir May, Daniel Novak

In the funding statement, ‘Brain Dynamics, grant number, CZ.02.01.01/00/22_008/0004643’ is listed incorrectly.

The correct funding statement is as follows: The study received funding from the Ministerstvo Zdravotnictví Ceské Republiky (Ministry of Health of the Czech Republic), grant number NV19-04-00233 (project CLIMABI); the Research Center for Informatics, Czech Technical University in Prague, grant number CZ.02.1.01/0.0/16_019/0000765; and České Vysoké Učení Technické v Praze (Czech Technical University in Prague), grant number SGS22/165/OHK3/3T/13. The funders had no role in study design, data collection and analysis, decision to publish, or preparation of the article.
