# Supplementary figures and images for: Statistical segmentation model for accurate electrode positioning in Parkinson’s deep brain stimulation based on clinical low-resolution image data and electrophysiology
Source: PLoS One. 2024 Mar 14;19(3):e0298320. doi: 10.1371/journal.pone.0298320 (PMC10939223; doi:10.1371/journal.pone.0298320)

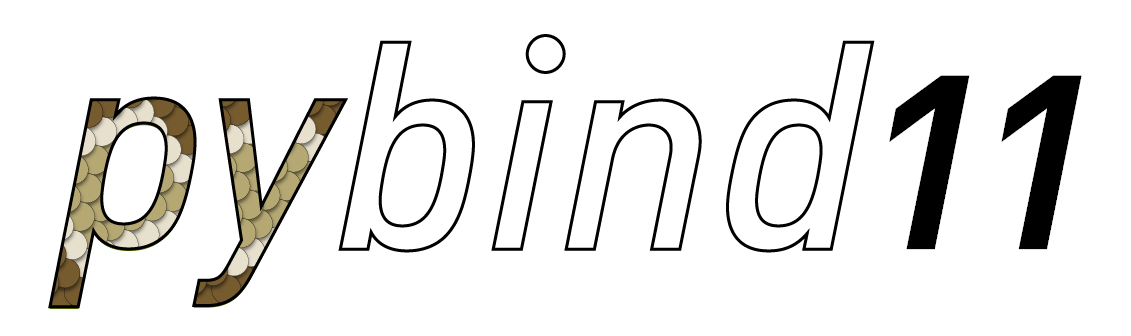

Supplement: S1 File — (ZIP) [file pone.0298320.s001.zip › bayessian_segmentation_cpp/external/pybind11-2.11.1/docs/pybind11-logo.png]

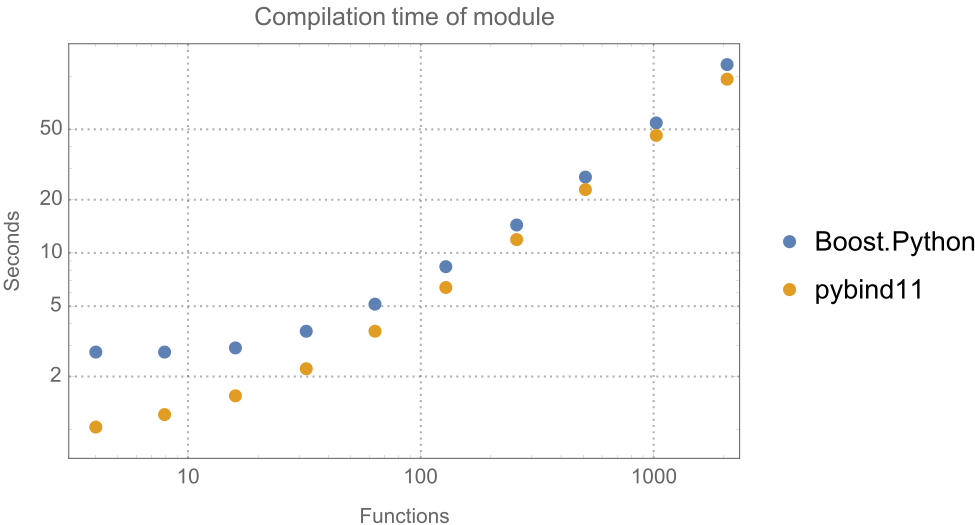

Supplement: S1 File — (ZIP) [file pone.0298320.s001.zip › bayessian_segmentation_cpp/external/pybind11-2.11.1/docs/pybind11_vs_boost_python1.png]

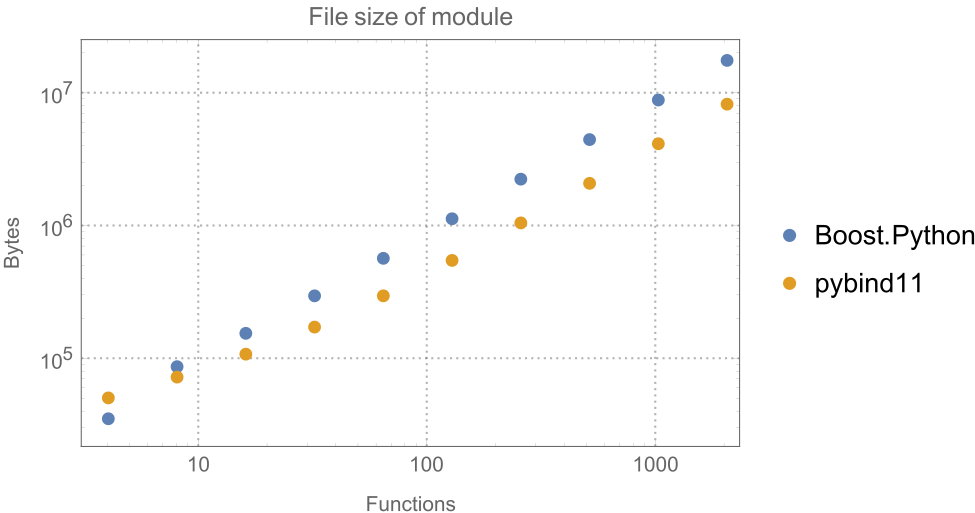

Supplement: S1 File — (ZIP) [file pone.0298320.s001.zip › bayessian_segmentation_cpp/external/pybind11-2.11.1/docs/pybind11_vs_boost_python2.png]
