# Supplementary material for: Statistical segmentation model for accurate electrode positioning in Parkinson’s deep brain stimulation based on clinical low-resolution image data and electrophysiology
Source: PLoS One. 2024 Mar 14;19(3):e0298320. doi: 10.1371/journal.pone.0298320 (PMC10939223; doi:10.1371/journal.pone.0298320)
Supplement: Attachment — Submitted filename: response_to_reviewers_and_editors.docx [file pone.0298320.s003.docx]

**Subject:**	Rebuttal letter on PLOS ONE submission PONE-D-23-24115

**To:**	John A. Thompson

Academic editor

PLOS ONE

Dear Dr. Thompson,

We are pleased to resubmit our revised manuscript entitled “*Statistical Segmentation Model for Accurate Electrode Positioning in Parkinson's Deep Brain Stimulation Based on Clinical Low-Resolution Image Data and Electrophysiology*” (PONE-D-23-24115). We are grateful to the reviewers and editorial board for providing insightful and thorough suggestions. We have addressed all of the reviewers’ and editorial remarks, some of which required substantial changes to the manuscript - notably, splitting the Results and Discussion sections, reworking or amending of most figures. Please find our comments below (in blue), reacting and summarizing the changes made for each of the reviewers’ or editorial suggestions.

UPDATE. Based on your confirmation request, we have contacted Prof. Mandar Jog for confirmation and found out that the correct point of contact for data related requests is the Institutional Review Board of the Western University. Thank you for helping us clarify this issue.

We are convinced the updates addressed the key concerns and increased the quality of the manuscript and ask you to re-consider the contribution for publication in PLOS ONE.

Best Regards,

Igor Varga

# Editorial remarks

**E1:** Please ensure that your manuscript meets PLOS ONE's style requirements, including those for file naming. The PLOS ONE style templates can be found at

We have checked the style guide and made appropriate edits in the main text, as well as in the references

**E2:** We note that the grant information you provided in the ‘Funding Information’ and ‘Financial Disclosure’ sections do not match.

Thank you for pointing out this discrepancy. We have checked the funding source numbers and provide the updated funding information below.

**E3:** Thank you for stating the following in the Acknowledgments Section of your manuscript:

We note that you have provided funding information that is not currently declared in your Funding Statement. However, funding information should not appear in the Acknowledgments section or other areas of your manuscript. We will only publish funding information present in the Funding Statement section of the online submission form.

Please remove any funding-related text from the manuscript and let us know how you would like to update your Funding Statement. Currently, your Funding Statement reads as follows:

“The funders had no role in study design, data collection and analysis, decision to publish, or preparation of the manuscript.”

We updated the acknowledgement section accordingly and removed the funding section from the manuscript. The funding statement should read:

“The study was supported by the grant of the Czech Ministry of Health, grant No. NV19-04-00233 (project CLIMABI), by the Brain Dynamics, grant number , CZ.02.01.01/00/22_008/0004643, Research Centre for Informatics, grant number CZ.02.1.01/0.0/16_019/0000765 and by the grant Biomedical data acquisition, processing and visualization, number SGS22/165/OHK3/3T/13., The funders had no role in study design, data collection and analysis, decision to publish, or preparation of the manuscript.”

**E4** We note that you have indicated that data from this study are available upon request. PLOS only allows data to be available upon request if there are legal or ethical restrictions on sharing data publicly. For more information on unacceptable data access restrictions, please see <http://journals.plos.org/plosone/s/data-availability#loc-unacceptable-data-access-restrictions>.

We have re-checked the availability of the dataset. The data is owned by a third party and unfortunately, we are not allowed to share the data in any way, including anonymized version, as there are legal restrictions on sharing data. The new data availability statement should thus read:

# “The study data is owned by Lawson Health Research Institute, London, ON, Canada, represented by Dr. Mandar Jog (mandar.jog@lhsc.on.ca). Sharing of the de-identified study data requires signing of a written data sharing agreement in compliance with the Ontario’s Personal Health Information Protection Act, the Research Ethics Board approved study aims (REB #109045, ReDA #2503) and written informed consent, signed by the participants.“

# Reviewers’ remarks

**Reviewer #1**:

In this study, the authors developed and tested software to automatically map the borders of deep brain nuclei including STN, automatically classify MER recordings as inside or outside STN, and correct for brain shift that results in mismatch between imaging and MER. They found 60% overlap between manual and automatic MRI segmentations and 85% accuracy between MER labeling and final STN modeling.

Main comments:

**R1.1:** - The authors have taken on a large task by attempting to develop both software for anatomic segmentation and MER fitting. A majority of the manuscript is dedicated to automatic STN segmentation which has been explored at length including using other methods which the authors also mention. The more interesting aspect is the classification and fitting of MER data to imaging which receives less attention in the manuscript.

Thank you for noting the imbalance in the manuscript. We have restructured the methods part, describing MER fitting, to be more in line with the structure of the MRI part and added more emphasis in the results and discussion sections. Although the MER part still occupies less space in the manuscript, we believe this is counterweighted by the references to our past papers, which describe parts of the respective MER-related methodology in more detail.

**R1.2:** The low sensitivity of the method is concerning. A relatively high specificity is to be expected given that most recording sites are not in STN.

We assume that the low sensitivity is connected to the discrepancy between the size of the MRI hypointensity, compared to the size of the electrophysiological STN. To develop on this topic, we added the following paragraph to the discussion section IV.D:

“*The relatively low sensitivity (correctly including the STN recordings) in all cases may be caused by the electrical field of the STN being dislocated, compared to the T2 MRI artefact, as previously reported by Bus(37) and more recently by Oxenford et al. (39). While the existing literature suggest a displacement of the MER-based and MRI-based STN, our results also suggest the electrophysiology-based STN to be larger. This is indicated by the scaling factors resulting from the MER-based fitting, which are often close to the pre-set constraint of 120% (see Fig. 10), as well as by the low sensitivity of the method. While our model works with smooth representation of the STN boundaries both in the MRI image, as well as in the MER, the determination of the hard boundary of the STN is a design decision, bound directly to the size of the resulting STN. For this initial feasibility evaluation of the proposed method, we decided to keep the resulting STN size close to that of the size of the T2 MRI hypointensity-based manual labels to limit the parametric space, at the sake of lower sensitivity. While future thorough evaluation of the overlap between T2 hypointensity and electrophysiological STN would be of high interest, higher quality MRI data would be needed to provide accurate estimates.*”

**R1.3:** As the authors acknowledge, the data set is limited by the low field strength (1.5 T) of the MR images and the inability to coregister postoperative CT imaging with preoperative MRI. This to some degree reflects the time at which the data were collected (2008-09). This does limit the interpretation of the results given that the data are of lower quality than current clinical data (generally using 3T MRI).

There was a typo in the original manuscript: the data were recorded between 2008 and 2019 in Western Hospital in Canada (2009 typo fixed in the methods section). The low field strength of the data used definitely contributes to the lower accuracy of the segmentation. While it is true that higher-field scanners are increasingly present at clinics across the world, we believe that a substantial proportion of clinics still recently used 1.5T imaging - E.g. an NHS report (2021) summarizes that in UK in 2021 still a majority of scanners in use had 1.5T field strength. The data recorded historically from 2008 and our primary focus was to achieve acceptable results on clinically commonly available low-resolution data. Moreover, the presented method is general and applicable to higher field-strength scanner, so the limitation mostly applies to the presented results, rather than to the method itself.

**R1.4** The authors do not discuss other authors that have attempted imaging-MER coregistration, particularly the creators of the LEAD-OR system (PMID: 35594135).

Thank you for pointing out the omission of some of the newest contributions. We reworked the methods and discussion sections and included the Lead-OR method as well in the following paragraph:

“*A similar task of combining microelectede recording with imaging data was approached in a recent study LEAD-OR (39), where the authors implement clustering of NRMS features and estimation of a brain shift with coregistration based segmentation approach with manual refinement. The LEAD-OR system focuses directly on intra-operative visualization and assessment and integrates with a clinical microrecording system to visualize the NRMS of the microelectrode recordings. and provides for visualization. While the authors also provide a coregistration-based segmentation of the subcortical structures, the authors did not provide segmentation based metrics as their pipeline included a manual refinementstep and therefore considered as a ground truth. The paper itself do not mention the scanning properties and quality of imaging data which is necessary to define accuracy of a measures. The authors also utilise clustering approach with NRMS values which is then merged with segmented boundaries. Compared to our approach, the LEAD-OR authors provided a simpler model, relying more on human input and corrections. On the contrary, we propose a fully automated method for MRI-based segmentation and fusion with MER – which may be a valuable future component of a system similar to the LEAD-OR.* ”

Minor comments:

**R1.5**

- The age range is listed as 38-75 years, and later the inclusion criteria are listed as 45-85 years; these are not compatible

Thank you for the comment. The range reported in the Data section of the methods incorrectly cited past version of a study protocol, which was later amended. We thus updated the figures to match the actual status of the dataset used.

**R1.6**

Typographical comments as requested by the journal:

- abstract sentence 1 - "neuropsychiatric disorders" should read "neurologic disorders" Fixed

- abstract sentence 2 - "suppress brain shift" should read "compensate for brain shift" Fixed

- introduction, paragraph 7 - "injecting multiple microelectrodes" should read "inserting multiple microelectrodes" Fixed

- methods, data subsection, paragraph 1 - please use past tense rather than a mixture of past and present tense Fixed

Thank you for pointing out the typos - we fixed all in the respective places.

**Reviewer #2:**

In this manuscript, Varga and colleagues report a novel segmentation model used to automatically define basal ganglia structures relevant to deep brain stimulation surgery. Using a leave-one-out validation, they demonstrate that the overlap between the automatically segmented nuclei and manually segmented nuclei is approximately 0.62 – 0.64 for the STN. Helpfully, this is compared to other reported models. Although other models have reported higher overlap (Dice) scores, there is certainly an element of clinical applicability here, as the mode**l uses only 1.5T MRI images,** whereas others have used higher intensity MRI (3T or 7T). Next, electrophysiology is incorporated into the model in the form of microelectrode recording data. The authors demonstrate that this strategy yields an overall accuracy of 85% for identifying the subthalamic nucleus, compared to the gold standard of a clinician-determined identification of location. The methods used to generate and test the model are rigorous and adequately explained. As such, this manuscript would likely make a novel contribution to the literature. I would suggest the following revisions:

Manuscript

**R2.1** Introduction: “Manual identification of STN and SN location from MRI images is the most common way to identify the most appropriate DBS target coordinates”. Either a citation should be provided for this statement, or the statement should be altered to include the use of indirect targeting, which is commonly employed for stereotactic surgery for PD.

Thank you for pointing out the omission: we changed the text to include indirect targeting methods in the literature as well.

**R2.2** Methods: Please include state the time between surgical implantation and the postoperative CT scan, as variation in this could lead to variability in the presence of intracranial air or other factors which could affect brain shift.

Unfortunately, we were unable to obtain detailed information on the time range in which the post-operative CT was provided, as this part of the co-registration and analysis was provided by a third party ( the Lawson health research institute). According to the available information, the post-operative CT scans were typically performed within a week from the surgery – suggesting that the effects of intracranial air, oedema etc may have contributed to the results as the reviewer suggests.

**R2.3** Results and Discussion, Section C. Several methodological steps not discussed previously are presented in this section. For example, the cluster analysis used to generate Figure 7 and the evaluation of electrode entry and exit points with respect to the STN are discussed here (and also in the figure legend for figure 8). These should be presented in the Methods section.

The description for figure 7 is described in Methods Section (C) (6) Surface-based statistics – which was originally not clear enough due to the differing titles and a missing reference. As we have now reorganized the methods section, according to other reviewers’ remarks, as well as modified the figure captions, we believe the description is now much easier to find.

**R2.4** In general, I would suggest separating the “Results and Discussion” section into separate “Results” and “Discussion” sections, in order to better clarify what is a result of the present analysis, and what is the authors’ interpretation of these results. Discussion of and comparison to other papers in this field should also be moved to the Discussion section.

Thank you for the suggestion, we have split the results and discussion sections and reorganized each into matching subsections for easier readability.

Figures and Tables

**R2.5** Figure 1 is somewhat confusing in its layout. It purports to show the evaluation stage for the trained model, but it is not clear to me that this is showing an evaluation method for the model. Instead, it seems to also show a schematic of the model’s inputs and outputs. I would suggest re-formatting this image to either more clearly show some sort of evaluation method or convert it fully to a schematic depicting how the model operates.

We modified the Figure 1 to more clearly depict the process of applying the pre-trained model to data of a new patient. We also modified the terminology and used “pre-training” to make a clearer distinction between fitting of probability parameters of the model (i.e. pre-training, done on a training data) and model application to a new patient’s data. We also changed the Figure 3 for graphical, as well as naming consistency and clearer overlaps.

**R2.6** Figure 5: Abbreviations used on the x-axis should be defined in the figure legend. The y-axis needs a label and definition of the units. The figure legend states that the STN was joined with SN for the segmentation, but the figure shows separate segmentation results for the STN and SN; this needs to be clarified.

Thank you, we added definitions of abbreviation to a figure legend of the Figure 5 and modified the y-axis label. As for the joint distribution of the STN and SNr structures, we modified the description in the text to be clearer. The idea is that the joint probability distribution can be used for fitting both the STN and SNr structures at once – as opposed to joining them to a single structure. This way, the method can utilise information about structural coalignment of these two structures, which have an unclear boundary in the T2 MRI imagery.

**R2.7** Table 1: Recommend adding interquartile ranges to the Dice scores reported with the SSM, to correlate with Figure 5. Abbreviations used in the table should be defined in the table legend. Also, the numbers 1 and 2 are used as annotations for footnotes, but do not correspond to any data marked “1” or “2” in the table.

Thank you, we have added the estimates of spread to Table 1 for our method, however, we opted for mean (standard deviation) format, which is internally consistent (both are parametric estimates), as well as consistent with mean values reported in the publications of other existing solutions, reported in the table.

As for table numbering – please see the response to your next remark.

**R2.8** There are two tables labeled as “Table 1”. The second one (i.e., Table 2) is not formatted well and overlaps with the body of the text.

This was apparently caused during the automatic conversion from MS Word to the PDF proof during submission, as the numbering was correct in the submitted word document. We now added manual table numberings to prevent this error.

**R2.9** Figures containing 3 dimensional representations of the STN (Figures 7, 8, 9) should have consistent axis labels and some description of the units. For example, the y-axis of Figure 8 ranges from 17-26, and the y-axis of Figure 9 ranges from 52-62. What are the units?

We have modified the axis labels in the figures, as well as the figure captions, to clarify the discrepancies. The discrepancy stems from the fact that the different figures use from principle different reference spaces (centered mean STN shape in mm in Figure 7, patient native MRI-based space in Figure 8 and different patient’s native MRI-based space in mm in Figure 9) – thank you for pointing out the discrepancies, we believe the additions made reading of the figures clearer.

**R2.10** - References Some references appear to be incomplete (e.g., missing journal information). These include references #24, 30. Reference 26 provides an archive website, but the original journal of publication is not cited.

Thank you, we have fixed the reference details.

**R2.11 Typos/**Minor issues

-Introduction: The reference for Zwirner et al., 2017 is provided in a different style from other references (i.e., all other references are numbered in the text). +

-Discussion: avoid using abbreviation “w.r.t” without first defining it. +

-Discussion, Section D, fourth paragraph: “ectrodes” should be “electrodes” +

-Figure 10: The figure caption appears to be cut off. +

-Limitations: There is a sentence which is cut off or incomplete (“We therefore stick to the”) Edited

Thank you, we have corrected the typos
